# Emergency Care Access Based on a Proposed CMS National Quality Measure

**DOI:** 10.1001/jamahealthforum.2025.0417

**Published:** 2025-04-11

**Authors:** Rohit B. Sangal, Craig Rothenberg, Richard Andrew Taylor, Arjun K. Venkatesh

**Affiliations:** 1 Department of Emergency Medicine, Yale University School of Medicine, New Haven, Connecticut; 2 Department of Biomedical Informatics and Data Science, Yale University School of Medicine, New Haven, Connecticut; 3 Department of Biostatistics, Yale School of Public Health, New Haven, Connecticut; 4 Center for Outcomes Research and Evaluation, Yale University, New Haven, Connecticut

## Abstract

This cross-sectional study uses a national electronic health record–derived dataset to characterize the proposed Centers for Medicare & Medicaid national quality measure for emergency care access.

## Introduction

Emergency department (ED) crowding has increased, straining hospital capacity nationwide.^1^ It has been attributed to increased inpatient boarding in the ED, resulting in delays in care access, extended lengths of stay (LOS), higher rates of patients leaving without being seen (LWBS), and worse clinical outcomes (eg, mortality).^2^ In response, the Centers for Medicare & Medicaid Services (CMS) developed the Equity of Emergency Care Capacity and Quality (ECCQ) hospital-level quality measure of access.^3^ Despite worsening access and disparities across sociodemographic groups, national benchmarks for accountability are lacking. We used data from a national electronic health record (EHR) system to apply the ECCQ measure and assess patterns in ED access failures over time and across patient subgroups.

## Methods

We performed a cross-sectional study from January 2017 to June 2024 using US data from an EHR system (Epic Cosmos; Epic Systems Corp) (eMethods in Supplement 1). Consistent with CMS methods, our primary outcome was the ECCQ score as a composite of 4 hospital access outcome failures: time to placement in an ED treatment space longer than 1 hour, inpatient boarding in the ED longer than 4 hours, total ED LOS longer than 8 hours, and LWBS. Failure of any component was considered a failure on the composite score for that patient.^3^ The ECCQ measure (score range, 0%-100%) is an inverse performance measure, expressed as the percentage of ED visits in which at least 1 of the 4 criteria is met, with higher percentages indicating a greater proportion of access failures. We describe overall ECCQ performance across the dataset and stratified by CMS-defined criteria, including age and ED visits for mental health conditions.^4^ Yale University Institutional Review Board deemed the study exempt from review because deidentified data were used. We followed the STROBE reporting guideline. Statistical analysis was performed with R, version 2023.06.01 (R Project for Statistical Computing).

## Results

In all, 148 618 000 ED encounters during the study period were included, with 27 188 408 encounters (18.3%) resulting in hospital admission. Overall, ED access worsened based on ECCQ performance from 18.5% in 2017 to 28.7% in 2024 (Figure) and among all 4 hospital access outcomes (Table). Prolonged ED length of stay (>8 hours) increased from 7.8% to 13.9%, and prolonged inpatient boarding (>4 hours) increased from 2.6% to 6.2%. Similarly, delays in placement into an ED treatment space (>1 hour) increased from 11.4% to 16.7%, while the proportion of patients who LWBS increased slightly from 0.8% to 1.1%.

**Figure.**
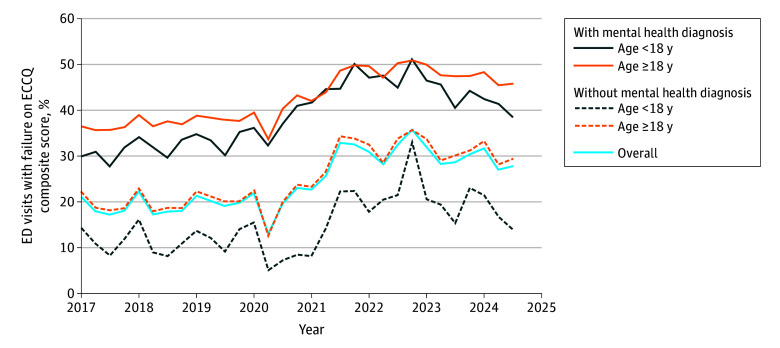
Emergency Department (ED) Visits With an Access Failure on the Equity of Emergency Care Capacity and Quality (ECCQ) Composite Score Stratified by Subgroup

**Table.** Characteristics of ED Visits During the Study Period According to the ECCQ Measure

Characteristic	ED encounters, No. (%)								% Change^a^
	2017	2018	2019	2020	2021	2022	2023	2024	
ED visits	19 061 060	19 564 168	20 046 451	16 525 366	18 111 009	19 486 198	20 149 972	15 673 776	NR^b^
Hospital admissions	3 211 778 (16.8)	3 387 769 (17.3)	3 541 400 (17.7)	3 250 184 (19.7)	3 516 208 (19.4)	3 547 135 (18.2)	3 735 047 (18.5)	2 998 887 (19.1)	2.3
ECCQ performance	3 534 398 (18.5)	3 676 484 (18.8)	4 021 244 (20.1)	3 272 843 (19.8)	5 199 245 (28.7)	6 201 710 (31.8)	5 995 423 (29.8)	4 503 755 (28.7)	10.2
**ECCQ performance stratified by hospital capacity outcome**									
ED LOS >8 h	1 481 707 (7.8)	1 574 727 (8.0)	1 753 138 (8.7)	1 652 639 (10.0)	2 490 628 (13.8)	2 854 631 (14.6)	2 848 649 (14.1)	2 176 888 (13.9)	6.1
LWBS	143 080 (0.8)	149 924 (0.8)	152 190 (0.8)	120 392 (0.7)	230 389 (1.3)	282 564 (1.5)	245 979 (1.2)	173 568 (1.1)	0.3
ED boarding >4 h	486 451 (2.6)	540 441 (2.8)	654 858 (3.3)	717 777 (4.3)	1 097 575 (6.1)	1 230 508 (6.3)	1 210 882 (6)	975 357 (6.2)	3.6
Arrival time to placement in an ED treatment space >1 h	2 173 381 (11.4)	2 212 731 (11.3)	2 404 977 (12)	1 686 148 (10.2)	3 040 367 (16.8)	3 812 087 (19.6)	3 566 332 (17.7)	2 620 287 (16.7)	5.3
**ECCQ performance stratified by patient age and presence of mental health diagnosis**									
Age <18 y with diagnosis	41 652 (30.3)	48 950 (32.4)	54 294 (33.5)	52 690 (37)	79 869 (45.5)	85 085 (47.7)	80 680 (44.3)	51 004 (40.8)	10.5
Age <18 y without diagnosis	438 362 (11.4)	432 713 (11.3)	479 747 (12.4)	264 811 (10.7)	539 682 (18.1)	908 167 (24.1)	735 284 (19.8)	480 765 (17.6)	6.2
Age >18 y with diagnosis	290 449 (35.9)	326 539 (37.3)	342 756 (38.1)	337 535 (39.2)	407 125 (45.9)	434 540 (49.3)	439 226 (48)	320 572 (46.4)	10.5
Age >18 y without diagnosis	2 763 935 (19.3)	2 868 282 (19.5)	3 144 447 (20.8)	2 617 807 (20.1)	4 172 569 (29.7)	4 773 918 (32.6)	4 740 233 (30.9)	3 651 414 (30.1)	10.8

Abbreviations: ECCQ, Equity of Emergency Care Capacity and Quality; ED, emergency department; LOS, length of stay; LWBS, left without being seen; NR, not reported.

^a^ From the first quarter 2017 to the second quarter 2024.

^b^ Percentage change was omitted because visit counts will increase as more hospitals are added to the electronic health record system.

ECCQ scores increased according to CMS criteria (age and mental health diagnosis). Among adults with and without a mental health diagnosis, access failures increased 10.5% and 10.8%, respectively. For pediatric patients with and without a mental health diagnosis, access failures increased 10.5% and 6.2%, respectively.

## Discussion

This study highlights challenges in hospital access captured by the ECCQ measure. Access failures were higher among patients with mental health conditions. Children without mental health diagnoses faced fewer access barriers.

Despite the belief that ED crowding is associated with increased demand for low-acuity ED visits, we observed increasing access failures despite total per capita ED use and hospital admissions remaining flat between 2019 and 2023 across the US.^5^ Instead, the decline in hospital capacity may be associated with challenges in postdischarge care transitions and prolonged inpatient stays, which may contribute to worsening ECCQ performance.^6^

The ECCQ measure, although limited to 4 hospital access metrics, highlights outcomes of insufficient acute care capacity. Each ECCQ component has been associated with care disparities,^3^ and we found that these inequities disproportionately affect adults and children with mental health needs. Efforts to improve hospital capacity should address long-standing structural gaps impacting all 4 outcomes, such as increasing mental health bed capacity.

A study limitation is reliance on data from a single national EHR vendor, which may not fully represent ECCQ performance across all hospitals. ECCQ’s integration into national reporting frameworks could be valuable for benchmarking performance, guiding quality improvement efforts, and improving emergency care access.

## Notes

Supplement 1.
eMethods

Supplement 2.
Data Sharing Statement
