# Supplementary material for: Emergency Care Access Based on a Proposed CMS National Quality Measure
Source: JAMA Health Forum. 2025 Apr 11;6(4):e250417. doi: 10.1001/jamahealthforum.2025.0417 (PMC11992599; doi:10.1001/jamahealthforum.2025.0417)
Supplement: Supplement 1. — eMethods [file jamahealthforum-e250417-s001.pdf]

Supplemental Online Content

Sangal RB, Rothenberg C, Taylor RA, Venkatesh AK. Emergency Care Access Based on a Proposed CMS National Quality Measure. *JAMA Health Forum.* 2025;6(4):e250417. doi:10.1001/jamahealthforum.2025.0417

eMethods

This supplemental material has been provided by the authors to give readers additional information about their work.

© 2025 Sangal RB et al. *JAMA Health Forum*.

eMethods

Data used in this study came from Epic Cosmos, a dataset created in collaboration with a community of Epic health systems representing more than 270 million patient records from over 200 healthcare organizations representing over 1000 hospitals from all 50 states and Lebanon. The current count values for patients, hospitals, and clinics are available on cosmos.epic.com

Data Representativeness

Cosmos is a robust dataset that encompasses a wide range of clinical and operational data, making it representative of the diverse population seeking healthcare in the United States. The organizations contributing data to Cosmos serve a broad patient demographic, enabling Cosmos to reflect the diversity of the U.S. population. Comparisons with the United States Census demonstrate that Cosmos provides a representative sample of healthcare-seeking individuals. (Statement adapted from https://cosmos.epic.com/about)

© 2025 Sangal RB et al. *JAMA Health Forum*.
