# Supplementary material for: Emergency Care Access Based on a Proposed CMS National Quality Measure
Source: JAMA Health Forum. 2025 Apr 11;6(4):e250417. doi: 10.1001/jamahealthforum.2025.0417 (PMC11992599; doi:10.1001/jamahealthforum.2025.0417)
Supplement: Supplement 2. — Data Sharing Statement [file jamahealthforum-e250417-s002.pdf]

Data Sharing Statement

Sangal. Emergency Care Access Based on a Proposed CMS National Quality Measure. *JAMA Health Forum*. Published April 11, 2025. doi:10.1001/jamahealthforum.2025.0417

Data
Data available: No

Additional Information
Explanation for why data not available: Data obtained and stored within Epic COSMOS and queries for data should be directed to to Epic COSMOS
